# Angio-Based Index of Microcirculatory Resistance for the Assessment of the Coronary Resistance: A Proof of Concept Study

**DOI:** 10.1155/2020/8887369

**Published:** 2020-10-25

**Authors:** Matteo Tebaldi, Simone Biscaglia, Domenico Di Girolamo, Andrea Erriquez, Carlo Penzo, Carlo Tumscitz, Gianluca Campo

**Affiliations:** ^1^Cardiovascular Institute, Azienda Ospedaliero-Universitaria di Ferrara, Cona, FE, Italy; ^2^Casa di Cura San Michele, Maddaloni, CS, Italy; ^3^Maria Cecilia Hospital, GVM Care & Research, E.S: Health Science Foundation, Ravenna, Cotignola, Italy

## Abstract

**Background:**

The study of coronary microcirculation has gained increasing consideration and importance in cath lab. Despite the increase of evidence, its use still remains very limited. QFR is a novel angio-based approach for the evaluation of coronary stenosis. The aim of our study was to use the QFR assessment in stable patients to recreate the IMR formula and to correlate the result of the two techniques.

**Methods:**

From June 1, 2019, to February 29, 2019, 200 patients with CCS and indication of coronary artery angiography and referred to the cath lab of the University Hospital of Ferrara (Italy) were enrolled. After baseline coronary angiogram, quantitative flow ratio, fractional flow reserve, and index of microcirculatory resistance evaluation were performed.

**Results:**

Pearson correlation (*r*) between angio-based index of microcirculatory resistance (A-IMR) and IMR 0.32 with *R*^2^ = 0.098, *P*=0.03: McNemar test showed a difference between the two tests of 6.82% with 95% CI from –12.05% to 22.89%, which is not significant (*P*=0.60). Bland and Altman plot showed a mean difference of 23.3 (from −26.5 to 73.1). Sensitivity, specificity, NPV, and PPV were 70%, 83.3%, 75%, and 70% for A-IMR value >44.2. The area under the ROC curve for A-IMR was 0.76 (95% CI 0.61–0.88, *P*=0.0003).

**Conclusion:**

We have validated for the first time the formula of the A-IMR, a tool for the calculation of microvascular resistance which does not require the use of pressure guides and the induction of hyperemia.

## 1. Introduction

Over the years, the study of coronary microcirculation has gained increasing consideration and importance in cath lab, both in stable and unstable patients [1, 2]. Despite the increase of evidence in favour of the study of coronary microvascular resistance (for example, with IMR), its use still remains very limited. The main factors that have limited its use are essentially (1) the induction of maximal hyperemia by adenosine in a critical patient setting such as that with STEMI, (2) the use of guides in patients without coronary stenosis, and (3) the increase in the procedural time. QFR is a novel approach for the evaluation of coronary stenosis significance based on 3-dimensional quantitative coronary angiography and contrast frame counting without the use of wire. QFR has shown good agreement with pressure wire-determined FFR measurements in patients with stable coronary artery disease [3]. The aim of our study was to use the QFR assessment in stable patients to recreate the IMR formula and to correlate the result of the two techniques.

## 2. Methods

### 2.1. Study Design

This is a single-center, investigator-driven, prospective study, which sought to validate the diagnostic performance of angio-based index of microcirculatory resistance (A-IMR), for the evaluation of the microcirculation resistances, using IMR as gold standard. All patients with CCS referred to the cath lab of the University Hospital of Ferrara (Italy) fulfilling the following criteria were eligible: (i) CCS with positive ischemia test and (ii) at least one coronary stenosis with a diameter between 40% and 90% at QCA analysis on left anterior descending (LAD). Exclusion criteria were (i) left main coronary artery diseases; (ii) multivessel diseases; (iii) extremely tortuous or calcified coronary artery; (iv) previous coronary artery bypass graft (CABG); (v) atrial fibrillation; and (vi) adenosine intolerance. The study was conducted in accordance with the ethical principles of the Declaration of Helsinki, and the protocol was approved by the institutional review board, and all patients provided written informed consent.

### 2.2. Study Procedure

Invasive coronary angiography was performed following the best local practice. After baseline coronary angiogram, quantitative coronary analysis (QCA, CAAS II, Pie Medical System) of LAD intermediate lesion was done with subsequent (i) QFR computation (software package QAngio XA 3-dimensional Medis Medical Imaging System, Leiden, the Netherlands, and (ii) fractional flow reserve (FFR) and index of microcirculatory resistance (IMR) evaluation. QFR, FFR, and IMR were obtained according to the method described previously [3, 4]. Cutoff values of abnormality were ≥25 for IMR and ≤0.80 for QFR and FFR. QFR, FFR, and IMR data were reviewed and assessed by two reviewers (SB and AE) in the core laboratory of the University Hospital of Ferrara in a blinded fashion. Cases of disagreement were resolved by consensus.

### 2.3. Angio-Based Index of Microcirculatory Resistance (A-IMR)

**Figure 1:**
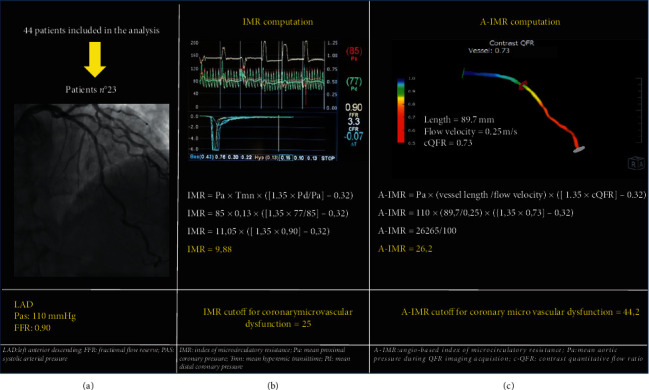
Example: (a) baseline characteristics; (b) IMR computation; (c) A-IMR computation.

The formula for the calculation of the IMR in the presence of coronary artery stenosis is as follows [5] (Figure 1):

(1) $$\begin{array}{c} \text{IMR}:\text{Pa}\times \text{Tmn}\times \left(\left[1.35\times \frac{\text{Pd}}{\text{Pa}}\right]\right)-0.32, \end{array}$$

where Pa = mean proximal coronary, Tmn = mean hyperemic transit time, and Pd = mean distal coronary pressure.

We can use the QFR assessment to calculate the IMR using these data:

Pa = patients mean aortic pressure that is available in the cath lab during QFR imaging acquisition.

Tmn = vessel length/flow velocity.

Pd = Pa × cQFR (contrast QFR value was obtained integrating the frame count analysis in the QFR computation.

In this way, the formula for calculating the cQ-IMR becomes

(2) $$\begin{array}{c} \text{A}-\text{IMR}=\text{Pa}\times \left(\frac{\text{vessel length}}{\text{flow velocity}}\right)\times \left(\left[1.35\times \frac{\left(\text{Pa}\times \text{cFQR}\right)}{\text{Pa}}\right]-0.32\right). \end{array}$$

If we simplify the ratio, we get the final formula:

(3) $$\begin{array}{c} \text{A}-\text{IMR}=\text{Pa}\times \left(\frac{\text{vessel length}}{\text{flow velocity}}\right)\times \left(\left[1.35\times \text{cQFR}\right]-0.32\right). \end{array}$$

We can then simplify the formula dividing the result by 100:

(4) $$\begin{array}{c} \text{A}-\text{IMR}=\frac{\text{Pa}\times \left(\text{vessel length}|/|\text{flow velocity}\right)\times \left(\left[1.35\times \text{cQFR}\right]-0.32\right)}{100}. \end{array}$$

### 2.4. Statistical Analysis

The present study is a prospective data collection. Thus, a formal sample size calculation is not applicable. Contemporaneously, for pilot studies, at least 30 patients are recommended [6].

Continuous data were tested for normal distribution with the Kolmogorov–Smirnov test. Normally distributed values were presented as mean ± SD and compared by *t*-test and one-way ANOVA. Otherwise, median (interquartile range), Mann–Whitney *U*, and Kruskal–Wallis tests were used. Categorical variables were summarized in terms of numbers and percentages and were compared by using the two-sided Fisher's exact test. Correlation and agreement between IMR and A-IMR were determined by the Pearson correlation coefficient (*r*), McNemar test, and Bland and Altman plot. To explore the A-IMR ability to identify microvascular disfunction (as identified by IMR), sensitivity, specificity, negative predictive value, and positive predictive values were reported, and receiver operating characteristics curves (ROC) with their area under the curve (AUC) were constructed. One- or two-tail tests were employed as appropriate, and the statistical significance was defined as *P* < 0.05. All analyses were performed with MedCalc 11.2.1 (MedCalc Software, Mariakerke, Belgium).by an independent statistician.

## 3. Results

From June 1, 2019, to February 29, 2019, 200 patients with CCS and indication of coronary artery angiography were referred to the cath lab. After the exclusion of 156 cases for technical and clinical meaning, the final population considered in the study consisted of 44 patients. 36 were male with a median age of 70 (44–85); 34 patients (77%) had hypertension; 26 (59%) had dyslipidemia; and 26 (59%) had diabetes. The mean cQFR, FFR, and IMR values were 0.88 [0.52–0.99], 0.87 [0.69–0.98], and 23.9 [7.8–57.3], respectively.

**Figure 2:**
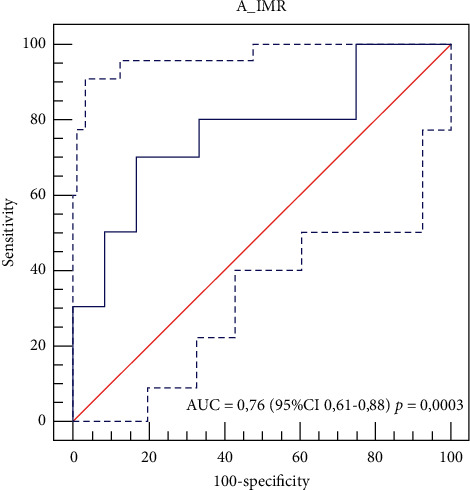
ROC of A-IMR vs. IMR.

Overall, Pearson correlation (*r*) between A-IMR and IMR 0.32 with *R*^2^ = 0.098, *P*=0.03: McNemar test showed a difference between the two test of 6.82% with 95% CI from −12.05% to 22.89%, which is not significant (*P*=0.60). Bland and Altman plot showed a mean difference of 23.3 (from −26.5 to 73.1). Sensitivity, specificity, NPV, and PPV were 70%, 83.3%, 75%, and 70% for A-IMR value >44.2. The area under the ROC curve for A-IMR was 0.76 (95% CI 0.61–0.88, *P*=0.0003) (Figure 2).

## 4. Discussion

In this proof of concept study, we have validated for the first time the formula of the A-IMR, a tool for the calculation of microvascular resistance which does not require the use of pressure guides and the induction of hyperemia but is based on the data available from the computation of cQFR. Our results show a good correlation of this new index with the IMR.

These data, if confirmed in subsequent studies, offer several advantages:

1. Stable patients admitted for CCS in the absence of coronary artery stenosis: evaluation of the coronary microcirculation on the basis of the coronary angiogram allows us to have important and very quick data that impact on patient's quality of life, as indicated in the ESC guidelines [1].
2. Unstable patients admitted with STEMI: the assessment of the microvascular resistance in the territory of primary angioplasty without the induction hyperemia, allows us to have a rapid, procedurally safe, “wire-free” evaluation, which has important repercussions on the patient's prognosis [2].
3. It could become a routine tool in the modern cath lab, implementing the information of a “simple coronary angiography” and improving the therapeutic approach of our patients.

## 5. Conclusion

In the future, A-IMR may be a valid tool for the evaluation of microvascular resistance.

## Data Availability

The results are extracted from the database present at the University Hospital of Ferrara.

## Abbreviations

AUC: Area under the curve
CABG: Coronary artery bypass graft
CCS: Chronic coronary syndrome
COPD: Chronic obstructive pulmonary disease
cQFR: Contrast quantitative flow ratio
FFR: Fractional flow reserve
IMR: Index of microcirculatory resistance
QCA: Quantitative coronary angiography
QFR: Quantitative flow ratio
ROC: Receiver operating characteristics curves.
